# APPROACH e-PROM system: a user-centered development and evaluation of an electronic patient-reported outcomes measurement system for management of coronary artery disease

**DOI:** 10.1186/s41687-024-00779-9

**Published:** 2024-08-28

**Authors:** Andrew Roberts, Eleanor Benterud, Maria J. Santana, Jordan Engbers, Christine Lorenz, Nancy Verdin, Winnie Pearson, Peter Edgar, Joel Adekanye, Pantea Javaheri, Courtney E. MacDonald, Sarah Simmons, Sandra Zelinsky, Jeff Caird, Rick Sawatzky, Bryan Har, William A. Ghali, Colleen M. Norris, Michelle M. Graham, Matthew T. James, Stephen B. Wilton, Tolulope T. Sajobi, for the APPROACH investigators

**Affiliations:** 1 Faculty of Medicine & Dentistry, University of Alberta, Edmonton, Canada; 2 Department of Medicine, University of Calgary, Calgary, Canada; 3 Libin Cardiovascular Institute of Alberta, University of Calgary, Calgary, Canada; 4 Department of Community Health Sciences, University of Calgary, 3280 Hospital Drive NW Calgary, Calgary, T4B 4B2 Canada; 5 Cohesic Inc, Calgary, Canada; 6 Ward of the 21st Century, University of Calgary, Calgary, Canada; 7 School of Nursing, Trinity Western University, Langley, BC Canada; 8 Department of Cardiac Sciences, University of Calgary, Calgary, Canada; 9 Faculty of Nursing, University of Alberta, Edmonton, Canada

**Keywords:** Electronic patient-reported outcomes, Usability evaluation, Heuristic evaluation, Coronary artery disease, Patient engagement

## Abstract

**Background:**

Coronary artery disease (CAD) confers increased risks of premature mortality, non-fatal morbidity, and significant impairment in functional status and health-related quality of life. Routine administration of electronic patient-reported outcome measures (PROMs) and its real time delivery to care providers is known to have the potential to inform routine cardiac care and to improve quality of care and patient outcomes. This study describes a user-centered development and evaluation of the Alberta Provincial Project for Outcomes Assessment (APPROACH) electronic Patient Reported Outcomes Measurement (e-PROM) system. This e-PROM system is an electronic system for the administration of PROMs to patients with CAD and the delivery of the summarized information to their care providers to facilitate patient-physician communication and shared decision-making. This electronic platform was designed to be accessible via web-based and hand-held devices. Heuristic and user acceptance evaluation were conducted with patients and attending care providers.

**Results:**

The APPROACH e-PROM system was co-developed with patients and care providers, research investigators, informaticians and information technology experts. Five PROMs were selected for inclusion in the online platform after consultations with patient partners, care providers, and PROMs experts: the Seattle Angina Questionnaire, Patient Health Questionnaire, EuroQOL, and Medical Outcomes Study Social Support Survey, and Self-Care of Coronary Heart Disease Inventory. The heuristic evaluation was completed by four design experts who examined the usability of the prototype interfaces. User acceptance testing was completed with 13 patients and 10 cardiologists who evaluated prototype user interfaces of the e-PROM system.

**Conclusion:**

Both patients and physicians found the APPROACH e-PROM system to be easy to use, understandable, and acceptable. The APPROACH e-PROM system provides a user-informed electronic platform designed to incorporate PROMs into the delivery of individualized cardiac care for persons with CAD.

**Supplementary Information:**

The online version contains supplementary material available at 10.1186/s41687-024-00779-9.

## Introduction

Heart disease, the second-leading cause of death in Canada, affects up to 8.5% of adults and accounts for an annual estimated cost of $21.2 billion [1, 2]. In addition to increased risks of premature mortality and non-fatal morbidity, coronary heart disease leads to significant symptoms and associated impairment in functional status and health-related quality of life [3, 4]. Effective patient-centered cardiovascular disease management should therefore include not only interventions that reduce mortality, but also aim to improve patients’ symptoms, functioning, and quality of life (QOL) [5].

There is growing evidence from the literature that the integration of patient-reported outcome measures (PROMs) and patient-reported experience measures (PREMs) into routine care can improve health outcomes and experiences with care, better align health services delivery with individual needs, promote patient-provider communication and support decision-making [6–12]. Although there are well developed and validated PROMs and PREMs for cardiovascular disease, there is a lack of electronic tools for collecting and reporting these measures back to care providers and patients. Recent advances have focused on electronic administration of PROMs in several other populations, including cancer [13, 14], orthopedics [15–17], mental health [18–19], stroke [20], and nephrology [21, 22]. Electronic platforms may ease the deployment of PROMs and enhance the ability to communicate information to patients and health care providers. In some settings, electronic PROMs/PREMs collection and reporting systems have yielded improvements in patients’ QOL, satisfaction, and clinical outcomes [12–15].

The Alberta Provincial Project on Outcome Assessment in Coronary Heart Disease (APPROACH) maintains one of the most comprehensive data repositories of CAD management globally [23]. It contains rich clinical information and outcomes for more than 300,000 patients who received cardiac care in hospitals in the Province of Alberta, Canada, since its inception in 1995 [24]. This registry has also collected patient reported information, including PROMs, using the 19-item Seattle Angina Questionnaire (SAQ) [25], the 5-item EuroQOL (EQ-5D) [26], the 18-item Medical Outcomes Study Social Support Survey (MOS-SSS) [27], and the 14-item Hospital Anxiety and Depression Scale (HADS) [28]. These PROMs have historically been mailed to patients with return postage within 1 week of their first coronary angiogram and at 1-, 3- and 5-years post angiogram. The completed paper-based PROMs are then scanned and uploaded into APPROACH. These PROMs data have been used in aggregate for research and reporting purposes. However, PROMs have not been deployed in routine cardiac care to facilitate communication with patients and promote shared decision making. Additionally, the financial burden of administering the paper-based PROMs is not sustainable. Consequently, we explored the transition of the PROMs data collection into an electronic format in which patients remotely self-report their health sta

tus and QOL via an electronic platform, and physicians receive the summarized information to support CAD management at the point of care.

The APPROACH electronic PROM system was developed in collaboration with patient research partners, cardiac care providers, heuristic and usability experts from the University of Calgary’s Ward of the 21st Century (W21C), Cohesic Inc. (an Alberta-based Information Technology vendor), and the Clinical Research Unit at the University of Calgary, which currently maintains the APPROACH clinical information system and patient-level registry data. In this study, we describe the development and usability evaluations of the APPROACH e-PROM system.

## Methods

### Stakeholders engagement

In September 2018, the APPROACH research committee, in collaboration with the Libin Cardiovascular Institute of Alberta’s patient advisory group, held an interdisciplinary workshop that focused on the use of PROMs and PREMs in cardiovascular care. This was organized to understand stakeholders’ perspectives about the relevance of PROMs, the appropriateness of the existing paper-based PROMs, and feasibility of migration of the paper-based administration to electronic administration format. Participants included 12 adults with lived experience with heart disease recruited from the APPROACH registry, cardiologists, decision-makers from Alberta’s Departments of Cardiac Sciences, and researchers with expertise in patient engagement and use of PROMs/PREMs in clinical care. Three small group sessions were held with representatives from each stakeholder group to identify gaps, research priorities, and potential solutions addressing the use of PROMs/PREMs information encountered in cardiovascular care. Conversations from these sessions were documented by designated research assistants who took notes from these sessions.

### Selection of PROMs for the APPROACH e-PROM system

A central aspect of the development of the e-PROM system is the selection of appropriate PROMs. We convened a working group composed of patient partners (NV, CW, WP, and PE), project investigators/ clinicians/cardiovascular researchers (CMN, MMG, SBW) and methodologists (TTS, RS), to review the appropriateness of paper-based PROMs for electronic deployment and use in routine clinical care. The working group developed recommendations for the set of PROMs to be implemented in electronic format. Factors considered included the type of outcome to be measured, the relevance of PROMs for assessing each outcome, the psychometric properties (validity, reliability, responsiveness to change, etc.) of each PROM, the burden on patient respondents to complete the instrument, and feasibility of integration of the results into clinician work-flow processes at the point of care.

### Technical development of the APPROACH e-PROM prototype

The APPROACH e-PROM system was designed in partnership with Cohesic Inc, to be a web-based electronic system accessible to patients via desktop and hand-held devices (i.e., tablets and smartphones). Other unique functionalities include inbuilt reminder notifications sent to patients to report outcomes at designated times, the capacity to administer multiple PROMs, and real-time delivery of reports to attending physicians in the form of an automatically configured 2-page report that summarizes the collected PROMs data on each patient. The initial prototype of this e-PROM system was developed and reviewed by the team of investigators and patient advisors.

### Heuristic evaluation of the APPROACH e-PROM system

Heuristic evaluation is a methodology for identifying design-related problems in interactive and dynamic human-computer interfaces by judging the interface’s design against a set of established standard guidelines [29]. It aims to identify design problems with user interfaces with the ultimate goal of making electronic health interventions easy to navigate for users. The heuristic evaluation of the APPROACH e-PROM system was conducted in collaboration with the University of Calgary W21C. Four heuristic evaluators, with expertise in user experience, user-centered design, data/information visualization, and human factors analysis were recruited through the network of experts available to W21C to evaluate the initial e-PROM system design features. Heuristic evaluation of the interactive patient-facing portal was structured around Nielsen’s principles for user-centered design [30]. The physician-facing static summary report prototypes were evaluated using Kelleher and Wagener’s principles of data visualization [31]. Each evaluator completed the heuristic evaluations independently and reported findings and recommendations in standardized spreadsheets. Once all evaluations were complete, a debriefing meeting was conducted for each evaluator to share their findings and answer any outstanding questions as needed. All findings and recommendations were collated into a single spreadsheet to inform the next design iteration of the e-PROM system.

### User acceptance evaluation of the APPROACH e-PROM system

Usability refers to “the extent to which a product can be used by specified user to achieve specified goals with effectiveness, efficiency, and satisfaction in a specified context of use” [32]. The acceptability of the e-PROM system to patients and physicians across web-based (personal computer) and hand-held devices (mobile phones, and tablets) was evaluated using user acceptance evaluations [33–35]. Patients with lived experience of CAD and those who may have previously completed the paper-based survey of the PROMs but had no prior experience with the e-PROM system were recruited to evaluate this tool. Eligible patient participants were recruited through our network of patient advisors and through the database APPROACH registry participants. Patient participants first completed a brief demographic questionnaire then completed the PROMs questions on the prototype of the e-PROM system either on a personal computer, tablet, or mobile phone. The participants’ confidence in completing assigned tasks on an electronic device was quantitatively scored using a single item 7-point Likert scale with response categories that range from 1(Not at all confident) to 7 (Very confident). Prompted by a standardized script, patients completed the e-tool while thinking aloud about their thoughts and actions. All sessions were recorded with permission. A researcher reviewed the sessions to make notes on patient verbal feedback, observed errors, or observed areas of difficulty. Notes were thematically analyzed to reveal insights on tool usability and barriers to use.

The physician-facing summarized PROMs report prototype was evaluated based on user acceptance and comprehension testing. The acceptability of the PROM summary report was conducted with 10 cardiologists involved in CAD management, recruited through the network of APPROACH clinician investigators. Using a standardized script, physicians were instructed to evaluate the e-PROM summarized report prototype while thinking aloud about their thoughts and actions.

The comprehensibility of the summary report was evaluated by asking the participants a set of questions designed to determine if they could reliably extract information from the report, and if the information extracted was correct. Three prototype summary PROMs reports were created. The comprehension test consisted of 30-minute one-on-one sessions with each participant, designed to solicit qualitative feedback, and to provide quantitative assessment of the ability of physicians to comprehend the data output of the tool. See page Appendix A1 for more details about the methodology to assess comprehensibility of the report.

All usability sessions were audio/video recorded for analysis. All issues encountered and feedback provided were noted by a research associate, qualitatively analyzed for themes, and summarized to provide recommendations for improvements to the report design.

## Results

### Stakeholders engagement

The interdisciplinary workshop with the stakeholders yielded important feedback with the following major themes. First, almost all participants agreed that the collection of PROMs is valuable and its integration into routine clinical care is central to promoting the delivery of patient-centered cardiac care to patients with CAD. Second, patient advisors identified individualized PROMs data used to inform decisions about disease management as a key priority. The delivery of a summarized PROMs report to patients and their care providers was deemed essential for a truly patient-centered care. Patient participants noted that existing PROMs did not capture some important aspects of patients’ quality of life. In particular, questions related to living alone, sexual function, sociodemographic and lifestyle factors were identified as important factors that may contextualize PROM results for attending cardiologists and facilitate patient-physician shared decision-making. Finally, the electronic formats for assessment and reporting of PROMs and PREMs were broadly acceptable and desirable to patients.

### Selection of PROMs for APPROACH e-PROM system

The PROMs administered via paper-based survey were considered too burdensome for some patients since it involved completing a total of 56 items from the four PROMs, in addition to other sociodemographic questions. Consequently, shorter versions of the PROMs were recommended to be adopted in the electronic system. For example, the 7-item SAQ [39, 40] and 8-item MOS-SS [41] scales were recommended as shorter versions of the 19-item SAQ and 18-item MOS-SS to achieve the right balance between minimizing respondent burden and capturing relevant PROMs information. Second, the HADS scale is not a sensitive measure for screening for depression in CAD populations [36]. It was recommended that this be replaced with the 9-item Patient-Health Questionnaire (PHQ-9) [37], as it has higher sensitivity and specificity values and positive likelihood ratio for screening for depression in CAD [37, 38]. Each item of the PHQ-9 is scored on a 4-point Likert scale that range from 0 (Not at all) to 3 (nearly every day), yielding a total score ranging from 0 to 27 with higher scores indicating more severe depression. In administering this instrument adopted a 2-step process for screening for depression using the PHQ instrument. In the first step, the 2-item PHQ (PHQ-2) was first administered to screen for depression while the full PHQ-9 is administered to those screen positive on the shorter version in the second step.Third, lifestyle self-management behaviors such as smoking, diet, exercise, and cardiac rehabilitation were recommended to be included in the form of 9 items relating to self-management on the Self-Care of Coronary Heart Disease Inventory (SC-CHDI) [42]. Finally, to facilitate the uptake of the summarized results, it was recommended that a two-page summarized PROM results should be presented with a high-level summary presented on the first and more granular details on each measure (e.g. subscale results on the second page). In summary, the 7-item SAQ, the 8-item MOS-SSS, PHQ-9, EQ-5D, and the SC-CHDI were adopted as the primary standardized PROMs for inclusion in the system.

### Heuristic evaluation

The four heuristic evaluators considered the e-PROM patient-facing portal to be well designed with relatively minor issues identified. Notable issues included; (1) the design of the progress bar, which did not help users gauge how far along they had progressed toward the end of the tool and how much was left to be completed; (2) the content of the survey questions (i.e., wording comprehensibility and consistency, spelling and grammar); (3) user control and freedom related to stopping and starting the survey; and, (4) issues related to navigation (i.e., forward and back) and response selection. The suggested adjustments were reviewed by our team of investigators and implemented by the technical team prior to beginning usability testing with patient participants.

For the summarized PROMs report, the evaluators identified issues with; (1) numerical placeholders, icons, legend design; (2) use of stacked bar charts for data visualization; (3) color coding of the trend icons, and other color choices; and (4) redundancy of information, and layout. One proposed solution to the identified issues included the use of a line graph with strip lines. This proposed solution supports the same tasks as the current designs, including learning the patient’s current and previous scores, understanding how the patient’s score has changed over time, understanding if the patient’s score is within a target reference range, and understanding if the patient’s score change is clinically significant. The APPROACH e-PROM system was revised based on these proposed changes.

### User acceptance evaluation

A total of 12 patient participants (5 female, 7 male) were recruited to evaluate the patient-facing e-PROM system across all devices. Of these, 5 completed the evaluation on their mobile phones, 5 on desktop computers, and 2 used tablets (Table 1). Participant’s ages ranged between 37 and 79. The average confidence rating (in easily completing tasks on an electronic device) is 6.0. Patient evaluators generally found the e-PROM system acceptable for its intended purpose Several participants also expressed appreciation for the thoroughness of the survey, and in particular the inclusion of the emotion-related questions:

**Table 1:** Distribution of patient participants’ characteristics for user acceptance testing of the APPROACH e-PROM system

Patients’ characteristics	Mobile	Tablet	Computer
Number of participants	5	2	5
Sex (% Female)	40	50	40
Age Range (years)	37–71	61–79	40–70
Confidence score* (Mean, Range)	5.5(4–6)	6.25(6–6.5)	6.4(6–7)

*NB* *On a scale of 1 to 7, with 7 being most confident, what is your level of confidence in working with an internet browser on a [select one: desktop computer/tablet/mobile phone]?

I really liked that on here you’re asking about the emotional side, that’s really good. I don’t know what you are doing about it, but somebody needs to ask.

I’ve filled out lots of surveys and none have asked questions like that.

It’s never any questions that a doctor would have asked me.

Feedback for refinement of the e-PROM system included technology-related concerns (in particular, text visibility issues on the mobile platform), physical implications (speculation that a patient lacking motor control or cognitive ability may have difficulty using the tool), as well as psychological considerations (including emotional state, particularly after depression screening questions, the ability to recall past experiences in order to accurately answer given questions, the desire for more system transparency when ending one survey and beginning the next, and overall willingness to complete the activity).

Some participants remained skeptical that their responses to the PROMs would be seen or used by the care providers in any sort of meaningful way. One participated stated:

You need to be in very rough shape to get the attention of your cardiologist… Waiting 14–15 months between appointments can be stressful, especially if you’re worried you have a problem.

**Fig. 1:**
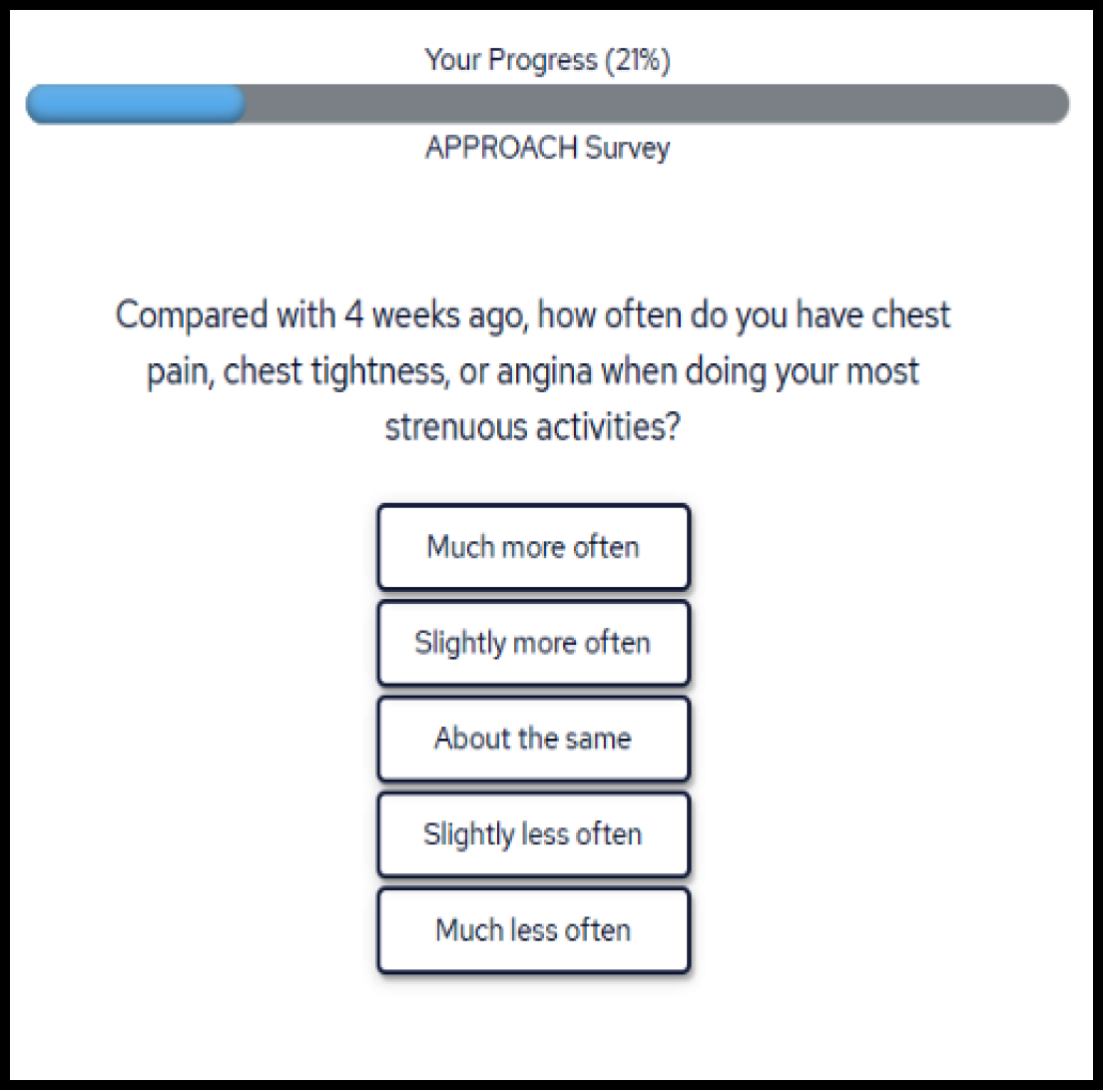
APPROACH e-PROM System patient user interface

Furthermore, some patient participants were unsure if their responses to questions about physical functioning should be based solely on their CAD symptoms or if their responses should include restrictions due to other physical comorbid conditions (e.g., arthritis). Participants suggested adding more specific instructions for how to respond to the PROM questions when other comorbid conditions affect their response(s). After reviewing this feedback, the APPROACH e-PROM system prototype was refined to include preamble information that prompts patients to interpret and respond the PROM questions in the context of their CAD disease. Figure 1 presents an example screenshot of the patient-facing interface of the APPROACH e-PROM for completing the PROMs.

The physician-facing PROMs summary report template was evaluated by 10 practicing cardiologists (3 females, 7 males). Physician ages ranged from 38 to 63 (average age 51), with an average of 17.4 years in cardiology practice and an average of 78.5% of time spent on clinical practice.

Participants reliably extracted correct information from the reports in nearly all cases (Table 2). The only notable errors were a mistake interpreting “below the 25th percentile” to mean a positive condition instead of a negative one; and some participants failed to notice supplemental information contained on the third and final page of some report prototypes (some reports only had two pages). Once familiarity is established with the report format, this is not anticipated to be an issue.

**Table 2:** Proportion (%) of physician participants who successfully interpret APPROACH e-PROM summary report

PROMs report	Task description	Report 1 (*N* = 4)	Report 2 (*N* = 6)	Report 3 (*N* = 5)
SAQ total score	How would you interpret this patient’s chest discomfort?	100	100	80
PHQ-2 total score	How would you interpret the result of this patient’s screening for major depression?	100	100	100
Equation 5D VAS	How would you interpret this patient’s overall quality of life?	100	100	100
SS	How would you interpret this patient’s level of social support?	100	100	100
SAQ Subscales	Drawing your attention to the SAQ-7 Subscales Results section, how are physical limitations, angina frequency, and quality of life contributing to this patient’s current health status?	100	100	100
PHQ-9 total score	What additional information does the report indicates about this patient’s likelihood of having depression?	50	N/A	80

*NB* SAQ-7 *EQ5D-VAS* 5-item EuroQOL Visual Analogue Scale; *PHQ-9* 9-item Patient Health Questionnaire; *SAQ-7* 7-item Seattle Angina Questionnaire; *SS* Medical Outcomes Study Social Support; *PROMS* Patient-reported outcome measures

Overall, the physicians had positive perceptions about the report with almost all respondents perceiving it as easy to understand. Several physicians lauded the visual appeal, its potential to provide an objective and quantitative assessment, especially for subjective symptoms, and its ability to provide an effective snapshot of a patient’s current condition and illness experience. The report was noted as a good tool to start a discussion with the patient, a “good communication tool”, and a helpful primer to prepare the physician to effectively direct the line of questioning during a consultation. The following are some illustrative quotes from the participants:

…the more information we have upfront that the patient is able to provide, the better job we can do.

I think this is a great tool and I would use it with every patient.

Visually it is attractive and easy to interpret.

I think it is pretty close to perfect.

This is wonderful. I would use it and I would encourage all my colleagues to use it. I think it’s a great time saver, and it also helps you establish that relationship with the patient.

The fact that this electronic system includes assessment of depression symptoms is very helpful. It does an assessment and gives you a recommendation in an area that we’re not as comfortable. That’s very useful.

Potential concerns raised by the physician participants included:

1. The potential to negatively affect the patient/physician relationship by partially replacing part of a traditional patient/physician encounter with an automated survey.
2. Increasing patient frustration by having them answer the same questions multiple times (i.e., in the survey, again for a resident or fellow, and again for the cardiologist).
3. Patients may be unwilling or unable to complete the lengthy assessment due to limitations with technical literacy, reading ability, language barriers, or physical impairments (blindness, lack of motor control, etc.).
4. Patients may be willing to complete the assessment once but may run out of patience if asked to complete it multiple times in a relatively short time interval (i.e., every six months).

Physician participants also expressed low confidence in interpreting the PHQ-9 measure, which was attributed to the ambiguity of the direction on how to manage or intervene on patients with mild depression (i.e., 5 ≤ PHQ-9 score ≤ 9). For example, the PHQ-9 scoring system recommends “watchful waiting” and/or repeated administration of the PHQ-9 at a follow-up visit for patients with mild depression. One participant stated:

I’m not sure if cardiologists are going to like that… it’s basically asking us to do more screening into their mental health status and I’m not sure that people feel comfortable doing that. It’s good to know they’re at risk, but they may not be seeing me again for a long time, nor am I the best person to assess or treat… That [sentence] leaves me feeling like I don’t know what I’m supposed to do.

**Fig. 2:**
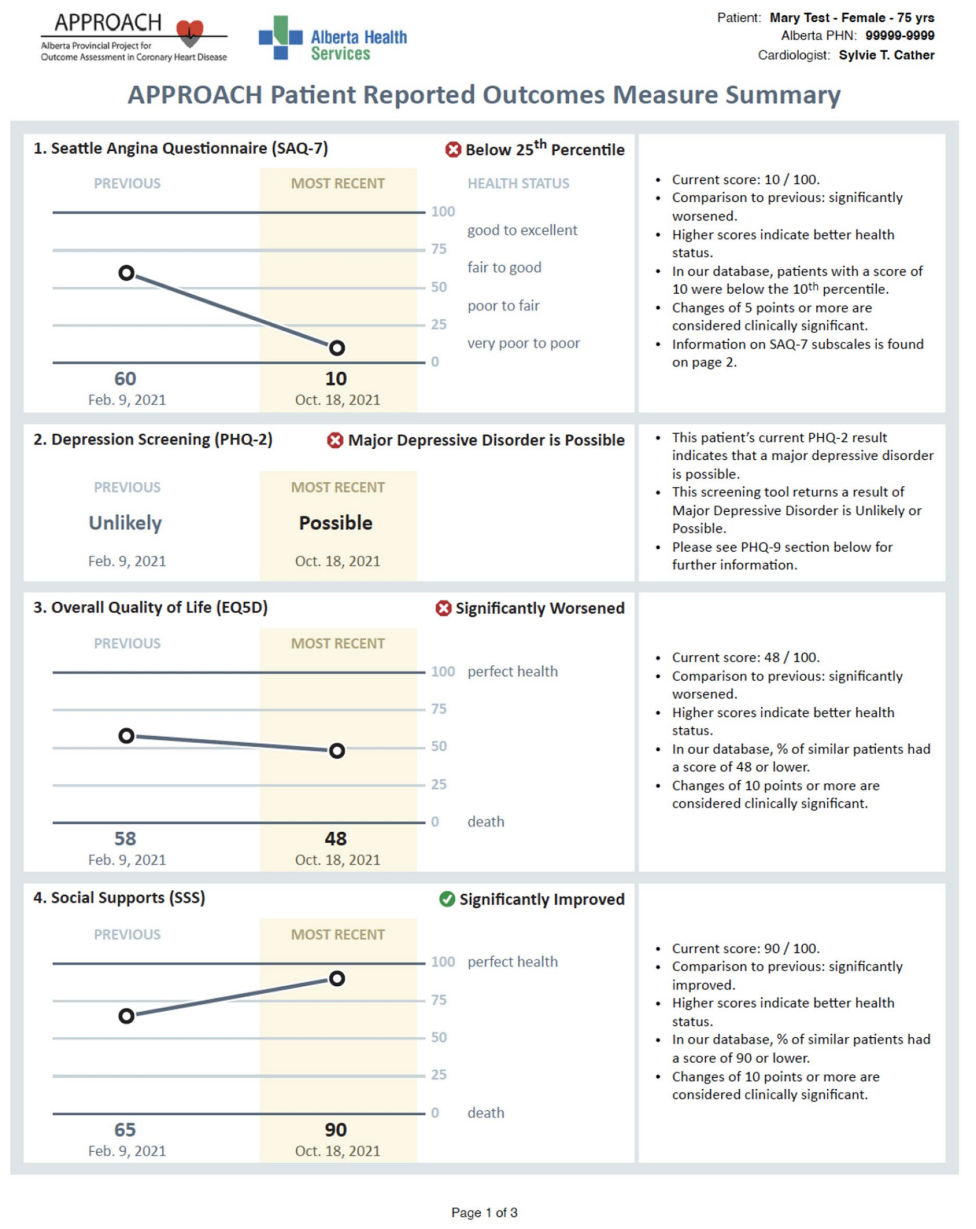
Physician-facing APPROACH e-PROMs Summary Report illustrating presentation of summarized information from the four PROMs instruments (First page). *NB SAQ-7* 7-item Seattle Angina Questionnaire; *PHQ-2* 2-item Patient Health Questionnaire; *EQ-5D* 5-item EuroQOL; *SSS* 8-item Medical Outcomes Study Social Support Scale

**Fig. 3:**
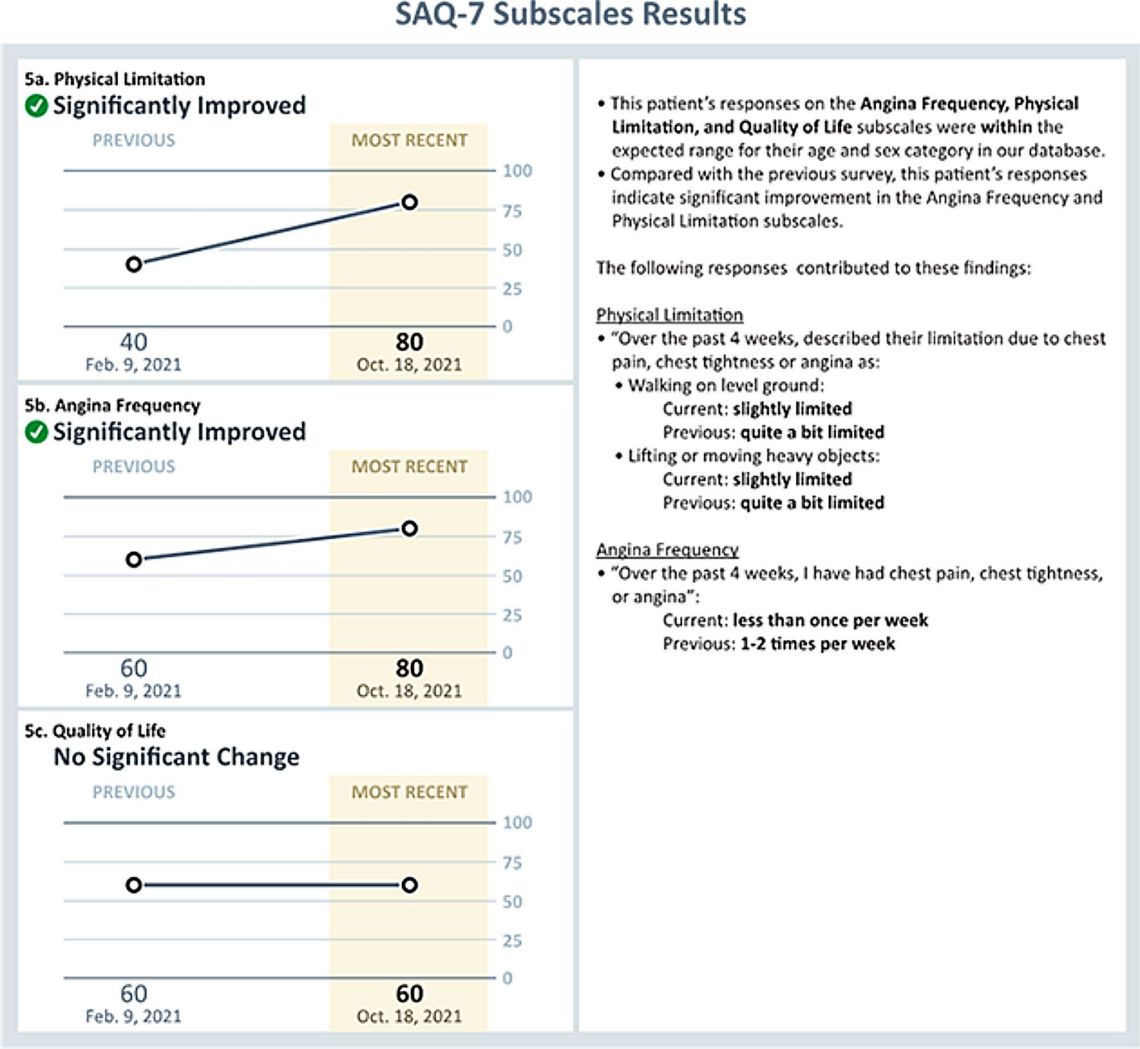
Physician-facing APPROACH e-PROMs Summary Report Illustrating Presentation of Information from the Subscales of the SAQ-7 (Second page)

**Fig. 4:**
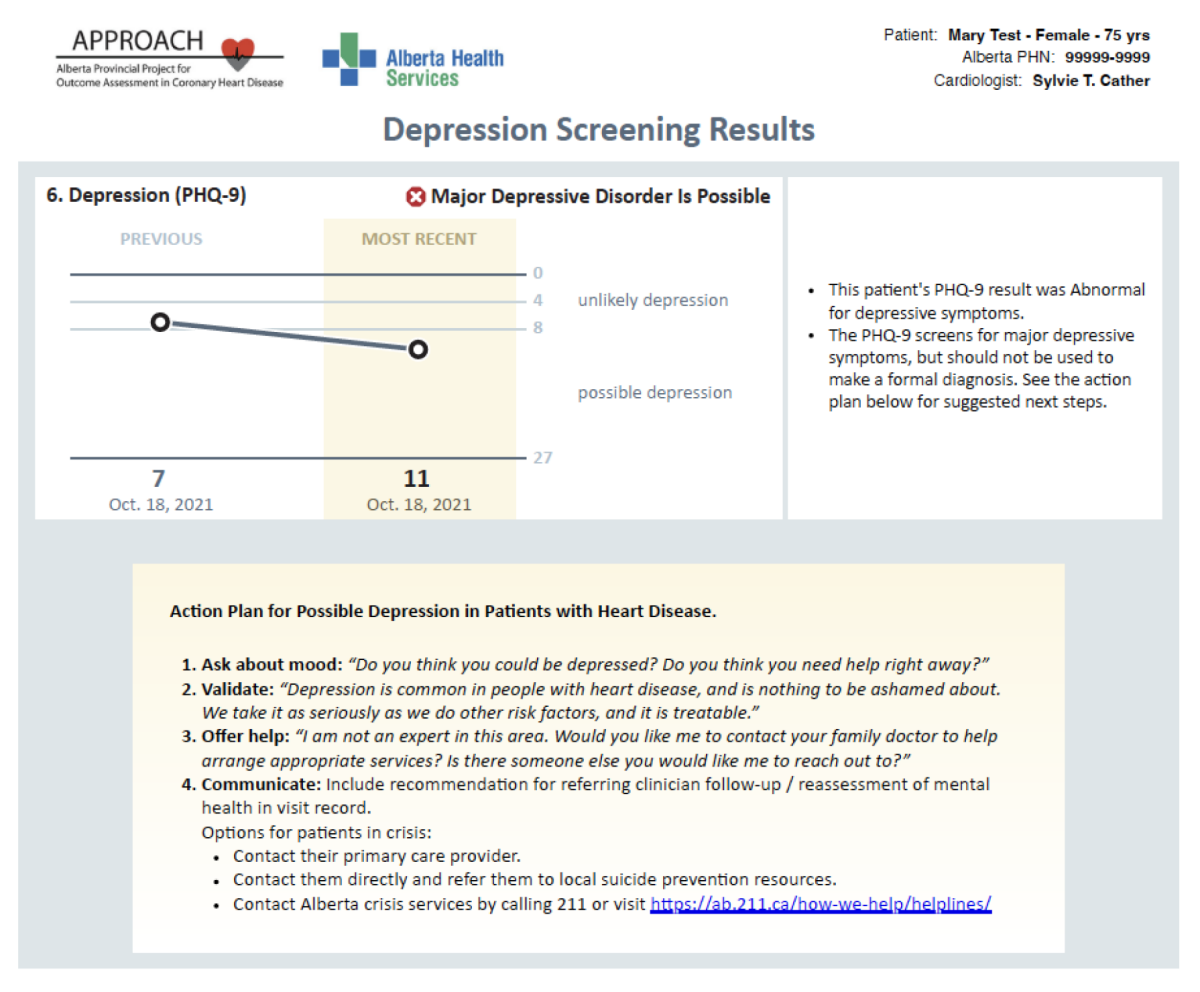
Physician-facing APPROACH e-PROMs Summary Report Illustrating Presentation of Information from the Subscales of the SAQ-7 (Third page)

To address this issue, we embedded additional information on relevant mental health and social support services, to which patients may be referred, in the summarized PROMs report to support physician decision-making. In Fig. 2, 3 and 4, we present screenshots from the final version of the summarized PROMs report after heuristic and user-acceptance testing.

## Discussion

The collection and integration of PROMs into routine clinical care to support clinical decisions has been identified as a key priority for effective cardiovascular disease management [43–44]. In this study, we describe the development and usability evaluation of the APPROACH e-PROM system for the collection of patient-reported outcomes in patients with CAD and the delivery of the summarized PROMs information to attending cardiologists to inform patient-care provider discussions during routine visits. Our study findings reveal that the APPROACH e-PROM system was perceived as easy to use, understandable, and acceptable to both patients and physicians. Although several electronic PROMs data capture systems have been implemented in routine cardiac care [45–48], the APPROACH e-PROM system is the first, to our knowledge, to explore the co-development of an e-PROM system with patients and to evaluate its usability for individuals with CAD.

An initial concern was the acceptability of an electronic system for completing PROMs in individuals with CAD, who are predominantly older adults. This is particularly important considering the electronic divide between older and younger populations. Our consultations and meeting with patients and members of the patient advisory group, revealed an overwhelming enthusiasm for electronic completion. This was further confirmed with the results of the usability evaluation, where patients rated the e-PROM system as acceptable and were confident in accessing and navigating its features from their electronic devices. However, we acknowledge that the number of patients included in the study was small, and the patients who participated in this study may not be representative of other patients encountered in clinical practice. Further research will be required to evaluate implementation and uptake of this e-PROM system among more diverse groups of people with CAD.

A recurring theme that emerged was the recommendation to deliver the PROM summary information to both the patient and the physician. There was hesitation about the feasibility of getting physicians to use PROMs results to enhance communication and shared decision making at the point of care, considering physicians’ busy workload. Delivering summarized PROMs s results directly to patients might further empower them to share concerns about their results with their cardiologists. This is consistent with evidence from the literature on the effectiveness of facilitated relay of information from patients to their health care providers [49]. Studies examining the effects of PROMs feedback have also shown improvements to decision-making, patient satisfaction, processes of care and some benefits to patients’ health outcomes [50–52].

Another notable finding is physicians’ limited knowledge on how to act on PROMs results relating to non-cardiac concerns such as mental health. To address this important barrier to meaningful use of PROMs in clinical care, we developed supplementary material in the form of guidance and links to resources for mental health problems and social support services embedded in the report. We have also developed an education program (providing continuing medical education credits) to encourage and support physicians to act on the PROMs results. This will be essential for the successful integration of PROMs into routine cardiac care.

The strengths of this study include its collaborative approach with patients, care providers, and researchers with interdisciplinary expertise who contributed to the user-centered design and development process of this e-PROM system. The demographic characteristics of our patient participants are representative of the general population of individuals living with CAD, confirming the generalizability of our study findings. The input from stakeholders, including patients, patient partners, care providers, cardiovascular researchers, methodologists, and user design and informatics expert were iteratively incorporated across the stages of design and development leading to iterative improvements in the prototypes evaluated in each step. We believe this iterative approach to design and development of the system improved the acceptability of the final system. A unique feature of this e-PROM system is the real-time delivery of summarized PROMs information to care providers at the point of care, in response to the recommendations from patient advisors about how PROMs should be used in the future, with a strong desire to see them used to inform decisions about their disease management and the need for the integration of the summarized information into clinical care. Evidence from previous literature reveals that the integration of PROMs in routine clinical practice has the potential to improve patient-care provider shared decision-making, disease management, and patients’ overall satisfaction with cardiovascular care [6–10]. The development of this e-PROM system will provide an opportunity for implementation of PROMS within clinical practice in Alberta to facilitate patient-care provider discussions and support clinical decisions about coronary heart disease management.

This study also has limitations. The development and evaluation of this APPROACH e-PROM system was impacted by the COVID-19 pandemic; most activities for this project were completed remotely due to the public health restrictions in place. This affected the recruitment of patients and physicians for in-person user acceptability evaluation of the APPROACH e-PROM system. Consequently, some of the patients completed their evaluation of the system remotely. Furthermore, our recruitment of patient participants for this study was limited to predominantly English-speaking white population. Our initial intension to ensure diversity of participants was severely inhibited as the COVID-19 pandemic disproportionately affected equity-deserving groups, such as racialized/visible minority groups and those from non-English speaking groups. Second, recruitment of 5 patients per electronic device was considered optimal for the usability evaluation of the e-PROM system user interfaces. This is at the lower end of the sample size recommended by the International Society for Pharmacoepidemiology and Outcomes Research [49]. Finally, the heuristic evaluation of the data visualization component of the APPROACH e-PROMs summarized report template using Kelleher and Wagener’s guidelines [31] proved to be difficult because this guideline was not applicable to the design of the data visualizations embedded within the PROMs summary report. Instead, we encouraged heuristic evaluators to provide their feedback and recommendations in a text file as appropriate.

Future research is required to evaluate the feasibility of implementing the APPROACH e-PROM system in selected cardiology clinics. The implementation of pilot study within clinical settings is needed to identify optimal approaches for broader prospective deployment of this e-PROM system into cardiac clinical care. Similarly, a randomized controlled trial is needed to evaluate its impact of an integrated e-PROM system in routine cardiac care on patient-physician communication, shared decision-making, processes and quality of cardiac care, and patients’ outcomes. This may be of particularly of interest to health services in jurisdictions where electronic clinical information systems exist, that can aid the collection and reporting of patient-reported data to health care providers. The APPROACH e-PROM system is designed with unique functionalities that allows for future seamless integration into electronic health record systems. Future research will evaluate the feasibility and the impact of this electronic system when deployed in routine clinical care across Canada to include diverse cultural and language groups. Additionally, while the initial goal for developing APPROACH e-PROM system is to improve individual patient and physician communication for optimal patient-centred CAD management, the ultimate goal of this initiative is to improve the processes of care and the quality of cardiac care delivery. Future research on e-PROMs systems should explore the potential effects on health system quality improvement and assurance initiatives. Finally, it is not clear to what extent are the psychometric properties to of the PROMs administered through the APPROACH e-PROM system is equivalent to the paper-based versions of these PROMs in the CAD population. Future psychometric investigations will be conducted to evaluate measurement invariance or equivalence of the PROMs administered digitally through APPROACH e-PROM system and the paper-based versions.

In conclusion, this study describes the development and evaluation of this APPROACH e-PROM system that is understandable and acceptable to patients and physicians and useful for routine collection PROMs and the integration of summarized PROMs information into cardiac care to inform communications between patients with coronary heart disease and their physicians. This feasibility of implementing this e-PROM system and its effectiveness within routine clinical care will be assessed in ongoing research.

### Electronic supplementary material

Below is the link to the electronic supplementary material.

Supplementary Material 1

## Data Availability

The datasets used and/or analyzed during the current study are available from the corresponding author on reasonable request.

## Abbreviations

APPROACH: Alberta Provincial Project for Outcome Assessment in Coronary Heart Disease Registry
CAD: Coronary artery disease
e-PROM: Electronic patient-reported outcomes measures
EQ-5D: 5-item EuroQOL questionnaire
PREMs: Patient-reported experience measures
HADS: Hospital Anxiety and Depression Scale
MOS-SSS: Medical Outcome Study Social Support Survey
PROMs: Patient-reported outcome measures
PHQ-9: 9-item Patient Health Questionnaire
PHQ-2: 2-item Patient Health Questionnaire
QOL: Quality of Life
SAQ: Seattle Angina Questionnaire
SC-CHDI: Self-Care of Coronary Heart Disease Inventory
