# Supplementary material for: APPROACH e-PROM system: a user-centered development and evaluation of an electronic patient-reported outcomes measurement system for management of coronary artery disease
Source: J Patient Rep Outcomes. 2024 Aug 28;8:102. doi: 10.1186/s41687-024-00779-9 (PMC11358368; doi:10.1186/s41687-024-00779-9)
Supplement: Supplementary file 1 — Supplementary Material 1 [file 41687_2024_779_MOESM1_ESM.docx]

**Supplementary Appendix**

**Physician User Evaluation Methodology**

The physician-facing summarized PROMs report prototype was evaluated based on user acceptance and comprehension testing. The acceptability of the PROM summary report was conducted with 10 cardiologists involved in CAD management, recruited through the network of APPROACH clinician investigators. Using a standardized script, physicians were instructed to evaluate the e-PROM summarized report prototype while thinking aloud about their thoughts and actions.

The comprehensibility of the summary report was evaluated by asking the participants a set of questions designed to determine if they could reliably extract information from the report, and if the information extracted was correct. Three prototype summary PROMs reports were created. The comprehension test consisted of 30-minute one-on-one sessions with each participant, designed to solicit qualitative feedback, and to provide quantitative assessment of the ability of physicians to comprehend the data output of the tool.

After informing the participant about the purpose of the study and receiving permission to record the session, the moderator used the “share screen” and “share control” functions in the Zoom video-conferencing program to share one of three randomly determined mock reports with the physician participant. The participant was encouraged to think aloud to share his or her first impressions of the report as well as where their attention was drawn, what questions arose, and what conclusions they could draw from the text and graphical presentation of measures. After a brief period allowing the participants to familiarize themselves with the report and to offer their first impressions and feedback, the moderator asked a series of questions to determine the ability of the participant to draw the correct conclusions from the mock report. If time allowed, participants were then shown a second example of a prototype report. Depending on how much time remained, these participants were asked for general feedback on the second report, or the comprehension questions were repeated.

**Summary of Feedback and Recommendation for Revision**

| Theme | Strengths | Recommendations for Revision |
| --- | --- | --- |
| Patient/Physician Relationship | *To have a tool like this [in clinic] would be unbelievable* | - Provide the patient with resources directly for things like anxiety, depression, or social isolation, instead of having to wait for resources to come through the physician. - Providing summary reports to patients would beneficial to patients, since they could see how their condition has changed, or how they compare to similar patients. - If the report may be viewed by patients, replace the large red “X” symbol with something less alarming. A negative or worsening result could be framed more positively as “an area with great potential for improvement.” |
| Effective Time | *“…The more information we have upfront that the patient is able to provide, the better job we can do...”*  *“…It’s a quick and effective snapshot…”*  *“…It could save time by not discussing areas that are not of concern…”* | - Information from the form could be auto-imported so that forms (for referrals, for instance) could already be pre-populated with the patient’s demographic information. - The report could be uploaded into the patient’s electronic health record as another item within clinical physician entry systems. |
| Decision-making | - This report helps the physician to consider the overall picture of a patient’s health when deciding if or how to intervene. | - In addition to chest discomfort, more cardiac symptoms (shortness of breath, heart failure symptoms, palpitations, presyncope, syncope) which are routinely checked should be included - Frailty scores was an additional parameter that could be included in the report. - Useful for decision-making in patients with valve disease and heart failure. - useful when used as part of an initial   assessment to get a quick overview of a patient’s history, and in particular the aspects that a  physician’s specific sub-specialty might not usually look at. |
| Depression | “That's very helpful. It does an assessment and gives you a recommendation in an  area that we're not as comfortable. That's very useful.” | - In order to make more effective use of the resources provided below the PHQ-9 section, - refer to it explicitly in the text section beside the graph (i.e., “for more information and resources, refer to the notes below”). - More explicit direction for “watchful waiting” is needed. The action should be to confer the information to the family doctor for follow-up. This action could be flagged more clearly, as it was missed by many participants. - Provide these resources directly to the patient automatically if depression is possible. - One participant suggested, “for watchful waiting, then, I would recommend some simple lifestyle things like going outside for a walk, make sure you're sleeping enough, eating well, yoga, meditation, that kind of thing.” These are examples of more tangible actions or suggestions the physician could use, which could be added to the resources section. |
